# Just Policy? An Ethical Analysis of Early Intervention Policy Guidance

**DOI:** 10.1080/15265161.2018.1523491

**Published:** 2018-11-26

**Authors:** Rose Mortimer, Alex McKeown, Ilina Singh

**Affiliations:** University of Oxford

**Keywords:** early intervention, justice, responsibility, children, parents, policy

## Abstract

Early intervention (EI) aims to identify children or families at risk of poor health, and take preventative measures at an early stage, when intervention is more likely to succeed. EI is concerned with the just distribution of “life chances,” so that all children are given fair opportunity to realize their potential and lead a good life; EI policy design, therefore, invokes ethical questions about the balance of responsibilities between the state, society, and individuals in addressing inequalities. We analyze a corpus of EI policy guidance to investigate explicit and implicit ethical arguments about who should be held morally responsible for safeguarding child health and well-being. We examine the implications of these claims and explore what it would mean to put the proposed policies into practice. We conclude with some remarks about the useful role that philosophical analysis can play in EI policy development.

Early intervention (EI) is receiving growing policy attention from UK governments, think tanks, and charities. EI aims to identify individuals or families at risk of poor health outcomes, and take preventative measures at an early stage, when intervention is more likely to be successful and cost-effective. As well as maximizing aggregate population health, EI is concerned with the just distribution of “life chances,” so that all children are given fair opportunity to realize their potential and lead a good life. EI policy design, therefore, invokes ethical questions about the balance of responsibilities between the state, society, and individuals in addressing inequalities and safeguarding child well-being.

In this article we analyze a corpus of EI policy guidance to investigate explicit and implicit ethical arguments about responsibility. Drawing on relevant debate within the bioethics literature, we examine the values underlying policy proposals, highlight where ethical principles conflict, and explore what it would mean to put the proposed policies into practice. While policy documents cannot be required to achieve the most rigorous standards of argumentation, we claim that lack of coherence will hinder effective translation from proposal into practice. Furthermore, we argue that unless we closely interrogate the underlying values and assumptions in ostensibly “obvious” policy initiatives, we run the danger of subverting the plurality of values and political outlooks held by the society in which policies are to be implemented. With this in mind, we conclude with some remarks about the role that philosophical analysis can play in EI policy development.

## BACKGROUND

We first provide a succinct historical summary of EI, outlining how the economic and ethical arguments that motivate such interventions have emerged in the United Kingdom. We then briefly explore some of the ways in which EI has developed internationally, particularly in the United States, in order to illustrate the relevance of our analysis to a U.S. readership.

### Early Intervention in the United Kingdom

The broad policy goal of improving lives of disadvantaged children through parent support has a long history in the United Kingdom. Training of specialist health visitors for young children began in the 19th century, and maternity benefit was introduced in 1911; these services fell under the Ministry of Health in the 1920s, before being subsumed within the National Health Service (NHS) after its creation in 1948. Dramatic social and cultural developments in the 1960s and 1970s created new challenges for those seeking to improve child welfare: changes such as the emergence of nontraditional family forms, and increasing numbers of women going to work (Bate 2017).

During this time, interventions designed to increase child welfare were a “dysfunctional patchwork of provision” (Bate 2017, 9). It was not until the Labour government of 1997 that these programs were organized into EI in the form that we recognize today. Sure Start Centres were first introduced in 1998, and in 1999 the UK government announced its target to eliminate child poverty by 2020. The publication supporting this declaration, “Opportunity for All: Tackling Poverty and Social Exclusion,” defined poverty not solely in financial terms, advancing a broader definition encompassing well-being and flourishing in the way that is now characteristic of EI policy guidance. The ensuing years of the Labour and subsequent coalition governments saw a steady proliferation of EI research and policy, some of which we analyze here.

Increasingly, these policy documents began to draw on findings in neurodevelopmental science and epigenetics that demonstrate the impact of a range of social stressors, such as structural inequality, neglect, and trauma, on the developing brain and on gene expression (Heim and Binder 2012; Johnson et al. 2016). These scientific findings are used by policymakers to advance the case for intervention in the 0–3 years period, since research identifies this as a critical “window” of development in which social stressors are especially impactful. For this reason, advocates of EI claim that intervention during the early years is both efficacious and cost-effective.

In response to the growing visibility and significance of EI research, policy, and practice, the Early Intervention Foundation was established in 2013, a core task of which is to evaluate various EI programs in terms of their effectiveness, and to make recommendations about “what works” to various commissioners, practitioners, and policy makers.

The vast majority of EI programs involve work with parents or pregnant women. Many programs take place in the family home, though others are held in outpatient clinics, children’s centers, or schools.^1^ There has been much debate concerning whether EI programs should be universal or targeted toward the most disadvantaged families; this is reflected in the history of Sure Start, which has moved between a targeted and universal model over its 20-year history (Bate and Foster 2017).

Though research has produced mixed results concerning the effectiveness of Sure Start in improving child outcomes, Sure Start paved the way for a proliferation of various other EI programs over subsequent years. With this came increased general acceptance of the idea that government should play some role in regulating and funding children’s development in the period from birth (arguably, from conception) to the start of school.^2^ In a post written for the London School of Economics blog, Naomi Eisenstadt writes:

The substantial success of the Sure Start scheme has been that the argument about the role government should play between birth and school is now won. … We no longer need to deliver more evidence that the pre-school years are vital to children’s development, and that provision of services for young children and families is critically important. … The acceptance that there should be provision for such services, and that government has a role in regulating and at least partly funding this, is now firmly in place. (Eisenstadt 2011)

However, despite the apparent certainty of the tone advanced by Eisenstadt, the extent, limits, and justifications for government intervention in family life are still very much up for debate, as our analysis will show.

### Early Intervention in the United States

The 1960s and 1970s also marked the beginning of the modern era in early childhood intervention in the United States. In 1963 the President’s Panel on Mental Retardation recommended the establishment of preschool programs in economically disadvantaged communities, designed to foster “the specific development of the attitudes and aptitudes which middle class culture characteristically develops in children, and which contributes in large measure to the academic and vocational success of such children” (quoted in Shonkoff and Meisels 2000, 14) This demonstrates how in the United States EI was closely tied with the need to address poverty and class-based inequality, and in this way parallels the UK EI agenda.

The first major EI program in the United States was Head Start, founded in 1964, nearly 25 years before the advent of Sure Start in the UK. Head Start was rooted in a belief in the crucial impact of early childhood experiences on later development; the program combined health, education, and social services, and worked hard to integrate parents both in the classroom and at the organizational level (Vinovskis 2008). In 1977, David Olds created the Nurse Family Partnership (NFP), another flagship EI program targeting “low-income” first-time mothers; these women are assigned a family “nurse” who visits regularly during their pregnancy, up until the child’s second birthday (Olds 2006). In 1996 the NFP received public funding from the U.S. Department of Justice, Office of Juvenile Justice and Delinquency Prevention, and since then it has substantially expanded, now operating in 42 states. In 2007, the UK government invested £7 million in “translating” the U.S. scheme for the UK context, creating the “Family Nurse Partnership,” which now operates in 90 sites across England, Scotland, and Northern Ireland (FNP National Unit 2011).

Today, scientific research—particularly neuroscience—lies at the heart of EI in the United States. In 2000, Jack Shonkoff co-edited the landmark report “From Neurons to Neighbourhoods: The Science of Early Childhood Development,” and in 2003 he co-founded the National Scientific Council on the Developing Child, a multidisciplinary, multiuniversity collaboration that seeks to recognize “the complementary responsibilities of family, community, workplace, and government to promote child well-being.”

This scientific argument for EI is combined with an economic case; the Harvard Centre on the Developing Child states that “society pays a huge price when children do not reach their potential.” The economic language used here echoes that of Graham Allen and other UK politicians who make the financial case for EI (see Allen 2011; Paterson et al. 2014; Davies 2013). Indeed, UK EI policy often draws from the work of U.S. economist Professor James Heckman, whose research on “the economics of human potential” envisions children as “human capital” (Kent 1988; Heckman 2000). In this way, both U.S. and UK policymakers engage with questions about what it means to create valuable (future) citizens, how science can be used to achieve this aim, and what responsibilities parents, families, and the state should be expected to take with regard to safeguarding child well-being.

### Ethical Questions

Given this background, EI research raises important ethical questions about the balance of responsibilities between the state, society, and individuals in addressing health inequalities, and the justifications and means used by policymakers to shape the cognitive, behavioral, and health outcomes of populations now and in the future. While such questions have been addressed within the social scientific literature (Macvarish et al. 2014; Meloni 2014; Pickersgill 2014), EI has received little bioethics scrutiny to date.

To address this gap in the literature, we conducted an ethical analysis of EI policy guidance documents published in the United Kingdom between 2006 and 2016. Through in-depth qualitative analysis of these documents, we excavate the normative claims, values, and arguments made both explicitly and implicitly within the EI policy literature. While the “good” of EI—improving the health outcomes of disadvantaged children—is often taken to be self-evidently right and just, our analysis closely interrogates this ostensibly “obvious” policy initiative. We examine the implications of the values in question, highlight where ethical principles conflict, and explore what it would mean to put the proposed policies into practice.

## METHODS

The first stage of document sourcing involved a general text search using www.gov.uk; search terms included, for example, “early intervention”; “early years” AND “prevention”; and “life chances” AND “families.” The text search was limited to documents published after January 1, 2006, and in total 32 documents were retrieved. This was supplemented by a text search using the Google search engine and the same terms; 14 additional documents were retrieved. The final stage of sourcing involved a snowball technique, following up references to other policy documents within those already gathered. This identified an additional 44 documents, bringing the total number to 100.

From these 100, we constructed a corpus of 17 documents. Corpus construction involves the selection a body of material, usually a collection of texts, to characterize the whole. Sample size is not important, as long as there is some evidence of saturation. Hence, our analysis began with one document—“Early Intervention: Good Parents, Great Kids, Better Citizens”—and others were added one by one through an iterative process until data saturation had been achieved. During analysis we continually moved back and forward between the documents, constantly assessing the corpus as a whole and considering whether new documents should be added in light of findings. Thus, while we began with one particular document, this did not determine the outcome of the analysis, since movement between documents was circular rather than linear. This said, in corpus construction, the selection of documents is “inevitably arbitrary to some degree [since] comprehensive analysis has priority over scrutiny of selection” (Atkinson et al. 2000, 23).

The final corpus was chosen to include a diverse sample of publishers, including various government departments, third-sector organizations, and think tanks. The heterogeneity of documents was deliberate; a diverse sample of organizations and government departments represents the range of perspectives on EI policy and reduces the potential for analytic bias inherent in a more narrow focus. Furthermore, EI is a cross-sector initiative in the UK. The final corpus shown in Table 1 is believed to constitute a characteristic sample of contemporary UK EI policy documents.

**Table 1.** Corpus of policy documents

Title	Author(s)/Editor(s)	Year of publication	Publisher
Building Character	Lexmond, J.	2009	DEMOS
	Reeves, R.		
Character and Resilience Manifesto	Paterson, C.	2014	The All Party Parliamentary Group on Social Mobility, with Centre Forum and Character Counts
	Tyler, C.		
	Lexmond, J.		
Character Nation	Birdwell, J.	2015	DEMOS, with the Jubilee Centre for Character and Virtues (Birmingham University)
	Scott, R.		
	Reynolds, L.		
Early Intervention: Smart Investment, Massive Savings	Allen, G.	2011	Independent Report to Her Majesty’s Government
Social and Emotional Skills in Childhood and Their Long-Term Effects on Adult Life	Goodman, A.	2015	University College London Institute of Education, on behalf of the Early Intervention Foundation, The Cabinet Office, and the Social Mobility & Child Poverty Commission
	Joshi, H.		
	Nasim, B.		
	Tyler, C.		
Fair Society, Healthy Lives. The Marmot Review.	Marmot, M.	2010	University College London Institute of Health Equity
Early Intervention: Good Parents, Great Kids, Better Citizens	Allen, G.	2008	The Centre for Social Justice and The Smith Institute
	Duncan Smith, I.		
Making Sense of Early Intervention	No named author	2011	The Centre for Social Justice
Our Children Deserve Better: Prevention Pays	Davies, S.	2012	The Department of Health, Her Majesty’s Government
The Best Start at Home: What Works to Improve the qçuality of Parent–Child Interactions From Conception to age 5 Years? A Rapid Review of Interventions	Axford, N., and colleagues	2015	The Early Intervention Foundation
The Early Years: Foundations for Life, Health and Learning	Tickell, C.	2011	An Independent Report on the Early Years Foundation Stage to Her Majesty’s Government
Opening Doors, Breaking Barriers: A Strategy for Social Mobility	Clegg, N.	2011	Her Majesty’s Government
Grasping the Nettle: Early Intervention for Children, Families and Communities	Davies, C.	2010	The Centre for Excellence and Outcomes in Children and Young People's Services (C4EO)
	Bromley-Derry, K.		
The 1001 Critical Days: The Importance of the Conception to Age 2 Period	Leadsom, A.	2015	A Cross Party Manifesto, in collaboration with The Wave Trust and the NSPCC.
	Field, F.		
	Burstow, P.		
	Lucas, C.		
The Foundation Years: Preventing Poor Children From Becoming Poor Adults	Field, F.	2010	Her Majesty’s Government
What Works to Enhance Inter-Parental Relationships and Improve Outcomes for Children?	Feinstein, L. (ed.)	2016	The Early Intervention Foundation
Social and Emotional Learning: Skills for Life and Work	Feinstein, L. (ed.)	2015	The Early Intervention Foundation

## ANALYSIS OF THE EI POLICY DOCUMENTS

Our focus is on one central ethical concept: responsibility. It should be noted that while EI policy is also concerned with the best interests of children, and thus engages with ethical questions about the meaning of a good childhood, our analysis found that these questions were secondary to the discussion of responsibility. EI policy engages with the issue of children’s welfare indirectly through a focus on the responsibilities of parents and the state. Thus, while an ethical discussion of the meaning of children’s best interests would be both interesting and important, such an analysis would not be true to the policy documents that we analyze here, and therefore we leave that task to future research.

Furthermore, we focus on responsibility—and relatedly, justice—because these concepts are tightly interwoven within both the goal of EI and the moral argument made in favor of it. A central aim of EI is to create parents capable of raising responsible citizens, who upon reaching adulthood will equally take responsibility for themselves and for their own children. Responsibility is also central to discussions of justice, which lie at the heart of EI policy; policymakers often claim that EI is a method for addressing inequality in society, but EI policy can only truly be just if responsibility for addressing injustice in society is fairly apportioned. Finally, policy should not only lay out arguments about what ought to be done, but also clearly state who should take action to create the required change. Therefore, a focus on responsibility allows us to interrogate policy claims in terms of their applicability and actionability in the real world. This lays the groundwork for the final section of this article, in which we discuss some of the ways in which philosophical bioethics can assist policymakers seeking to navigate a philosophically as well as politically complex moral landscape.

What follows is an outline of the key moral argument that drives UK EI policy; we then explore in more detail how responsibility is understood and configured across the corpus.

### Key Argument: Distribution of Life Chances Is Unjust

EI policy contains numerous examples to show that the distribution of life chances is unjust in contemporary society. The central argument is that much of a child’s future and “life chances” are shaped in his or her first 2 years; here, “life chances” means something like real opportunities to fulfill one’s potential and succeed. Since some babies do not have a good (or good enough) start in the early years, they do not have the same opportunity as others to lead a healthy, successful, flourishing life.

### Parental Responsibility in an Unjust World

The documents draw upon evidence from the neurodevelopmental sciences to demonstrate what or who is causally responsible for a “bad start” and subsequent inequalities. With one notable exception,^3^ the policy papers suggest that parenting is the most important cause of poor outcomes for children, far more impactful than social factors like inequality or poverty. Indeed, the documents go so far as to claim that good parenting can override almost all other kinds of disadvantage:

What parents do is more important than who they are. Especially in a child’s earliest years, the right kind of parenting is a bigger influence on their future than wealth, class, education or any other common social factor. (Allen 2011, xiv)

Poverty is a factor, but not a central one … I am fond of saying poverty of what? And actually it seems to be poverty of the parent–child experience … that leads to poor child outcomes rather than poverty of a material kind. (Scott 2009)

Agency emerges as an important consideration within the guidance; the documents often suggest that determining whether an individual had sufficient power to do otherwise when bringing about a specific outcome is essential if they are to be candidates for praise or blame. In keeping with this emphasis on agency, the documents do not always equate causal and moral responsibility; this is consistent with much of the philosophical discussion of responsibility, which sees causal and moral responsibility as distinct yet related concepts. Generally speaking, to be morally responsible for something is to be worthy of a particular kind of reaction—blame, or sometimes praise—for having performed it (on blameworthiness, see Coates and Tognazzini 2013, §2.1; Kenner 1967). Thus, moral responsibility requires agency and personhood. Causal responsibility, in contrast, requires only a causal link between two states of affairs, and need not be attributed to persons with agency. For example, it makes sense to say that the rainfall caused the flood, or the dog jumping caused the vase to smash, but in neither case does it make sense to attribute moral responsibility.^4^

### Moral Responsibility and Blame

While the documents attribute causal responsibility to parents, relying on empirical data to justify their position, they are often reluctant to blame parents on the basis that many parents could not do other than care for their children in the way that they do; indeed, this is why EI is required:

We do not blame—they [parents] are to a large extent the product of their childhoods as, in turn, were their parents and grandparents. (Loughton 2015, 14)

In support of this position, the documents often adopt a stance that parents are unable to act differently because they do not know better:

Parenting style is a learned behaviour—it is “a product of parents’ own experiences and education”. In effect, information as to the behaviours most likely to aid child development is unevenly distributed and, as such, these behaviours are unevenly practised. (Paterson et al. 2014, 26)

Here bad outcomes are caused by a lack of education; the claim made is that in some families, information about the best parenting styles is not passed down to children. This creates a vicious cycle wherein parents model child care on their own experience of being parented and, perhaps unknowingly, perpetuate bad outcomes. They should not be held morally responsible for their parenting, because their lack of knowledge made it inevitable that they would do so.^5^ Further, the corpus often suggests that parents are impacted by poor parenting in the early years of their own lives in ways that give rise to dispositions over which they have little or no control; this might include mental health problems such as depression, or “undesirable” temperaments such as low confidence, lack of empathy, or inability to delay gratification. Thus, parents are not only unable to do otherwise, but also unable to be otherwise, absolving them of moral responsibility.

Given widespread social biases against “bad” parents, it is noteworthy that some of these policy documents try to protect parents from blame. However, this position can also be interpreted as paternalistic, since it views parents as has having little or no agency to formulate parenting goals and to effect change. Further, if we consider how such claims might be translated into practice, this reveals limitations typical of those encountered in any instance where the ideal precepts of a theory are imposed upon nonideal real-world circumstances.

### Blame, Praise, and Moral Luck: From Policy to Practice

First, the nature and extent of “power to do otherwise”—responsibility—is uncertain both empirically and theoretically. No incontestable evidence or argument shows whether or not people act freely (Kane 2011). Are we responsible for acting in ways that are influenced by our earlier circumstances, or are we not?

One might argue that since it is unjust to punish people for being in situations over which they have no control, so it is unjust to punish people for actions that they commit because of a character formed by an upbringing over which they had no control. This appears to be a neat theoretical solution that sidesteps being forced to endorse undue punishment. It has troubling implications in the EI context, however. Adopting this stance leads to a regress, since it makes the apportioning of blame to one’s parents arbitrary, given that they too had parents who brought them up in a way over which they had no control. Practical ethical problems may arise, therefore, in basing EI policy on the principle that individuals should not be held morally responsible for causing harm resulting from aspects of their character formed by earlier life circumstances over which they had no control. It is problematic not only for determining who is responsible, but also because it risks treating victims unjustly. Do we really want to say, for example, that children are not entitled to hold their parents responsible for bringing them up badly?

The reluctance to apportion moral responsibility also produces unwelcome conclusions in relation to behavior that we deem desirable. The commitment to a no-blame position threatens to undermine our ability to reward good behavior; indeed, this is one of the criticisms often made against proponents of Luck Egalitarianism. Luck Egalitarians hold that inequalities are unjust when they result from “brute luck”—such as being born to poor or inept parents—yet just when they result from factors that an individual can control, like informed choices or hard work (Arneson 2004). But if we cannot hold individuals responsible for poor conduct because their actions were made probable by earlier conditioning beyond their control, then presumably we cannot reward individuals lucky enough to be brought up in circumstances that make it more likely that they will act in ways of which we approve.^6^

The corpus seems reluctant to take this step; policymakers wish to avoid blaming the unlucky for their failures, but are willing to praise the fortunate for their successes. To make this position appear consistent, the line between what is and is not the result of luck becomes increasingly blurred, as is the case in the following declaration of the Clegg report:

No one should be prevented from fulfilling their potential by the circumstances of their birth. What ought to count is how hard you work and the skills and talents you possess, not the school you went to or the job your parents did. (Clegg 2011, 5)

Apparently, then, how hard one works or the skills and talents one possesses are not features of one’s character determined by the circumstances of one’s birth. However, a consistent message of the policy corpus is precisely the opposite: EI is only viable if behavior and character can be influenced by taking particular identifiable courses of action in the early years. The preceding quote suggests that being skilled and hardworking is deserving of reward, and yet a significant part of having such a character is determined by the circumstances of one’s birth, over which one had no control. By contrast, adverse circumstances early in life make it less likely that one will develop in the way that the policymakers deem worthy of reward, and of course we can agree with the Luck Egalitarian’s normative claim that one ought not to be held back by being born into such circumstances (and, indeed, that we ought to find ways to ensure that people are not).

This contradiction confuses EI policy, as it is not clear who should be held responsible for what outcomes, and what should be done to address injustice. Despite the apparent moral certainty of the position laid out in support of the policy, under analysis it provides no consistent or clear answer to these questions. This ambiguity creates difficulties concerning how policy ideals would be translated into practice.

### Inequality and Aspirational Parenting

While the policy corpus resists blaming “bad” parents, it advances the view that good parenting is key to achieving equality of opportunity, by ensuring that all children receive the best start in life. In particular, parents who are aspirational, hard working, and selfless are praised. Thus, the corpus rests on normative assumptions about the meaning of good parenthood and the values inherent in family life. However, these assumptions cannot be taken to be self-evident. For example, Swift (2004) advances a theory of “legitimate parental partiality” whereby parents may engage in some activities that benefit their children, like reading bedtime stories, but not others, like buying them access to a better education through enrollment at an elite private school. The distinction between what is and is not permissible stems from Swift’s account of the goal and purpose of the family. He argues that certain vital relational goods are realized through close, loving familial connections, and it is acceptable for parents to pursue these goods even though less fortunate children will not have access to them. However, the purpose of the family is not to function as a social network designed to grant competitive advantage to its members, Swift argues, and as such parents have a responsibility to abstain from pursing advantages for their children when doing so perpetuates inequality and harms those less fortunate, without securing important relational goods in the process.

Thus, policies that insist on aspirational parenting as a cure for inequality fail to consider the ways in which aspirational parenting may promote unfair advantage and increase inequality. Of course, policymakers can maintain their position without contradiction by claiming that if all parents were sufficiently aspirational, then all children would fare better, even if relative inequality were not reduced; this would be a scenario acceptable to priority or sufficiency accounts of justice.^7^ Furthermore, policymakers might want to advance a view of the family that is quite different from Swift’s, in which good parents do seek to give their child a competitive advantage over other children, where they are concerned not with adequacy but with achieving the best. We do not endorse a particular view of “the good family” here. Rather, we highlight possible implications of the policy claims, and the implicit values that underlie them.

### Parental Versus State Responsibility

There is general agreement across the corpus that parents are assumed to be responsible for child well-being in the first instance, since in the absence of information to the contrary they have sufficient power to create good outcomes for their children regardless of social factors. This is sometimes stated sufficiently boldly that it appears to relieve the state of responsibility:

The quality of this [early years] nurturing has a major impact on how well children develop and then fulfil their potential. This task is not primarily one that belongs to the state. We imperil the country’s future if we forget that it is the aspirations and actions of parents which are critical to how well their children prosper. (Paterson et al. 2014, 6)

However, as we have shown, sometimes parents are prevented from enacting their responsibilities as a result of their own experiences of received poor parenting; a vicious cycle of poor parenting develops from which people are often unable to break free without assistance. At this point, the corpus generally agrees that the state has a responsibility to act, deriving from its power to implement changes, and the potentially serious adverse consequences of allowing inadequate parents to take on the critical task of raising children:

As a society, we seem to have reduced the standards of responsibility which we expect parents and households to meet when children are born. This has produced a tacit acceptance (particularly from those who do not have to face the consequences) of many of the dysfunctional conditions least favourable to successful child-rearing. (Allen and Duncan Smith 2008, 30)

Here, a distinct ethical position is taken, grounded in the vulnerability of children and the duty to protect them from harm. The quote suggests that “we,” presumably society, should not accept poor parenting. A strong interpretation of this claim might be that only parents who are able to meet a high standard of responsibility should be allowed to raise children. Philosophers and bioethicists have explored the implications of such an extreme position; for example, LaFollette (1980) advocates licensing parents in the same way that one provides driving licenses to those who pass a test; similarly, Cassidy (2006) suggests that only the minority of people who can parent excellently should have children, and the rest of society should dutifully abstain from doing so to prevent harm to one's potential offspring.

If these were policy proposals rather than thought experiments, they would likely be un-implementable.^8^ Society would deem them an unreasonable infringement of an individual’s right to a family, irrespective of the strength of any case for EI to ensure that children are not harmed by poor parenting. The architects of the policy document quoted here are unlikely to endorse such an extreme interpretation; however, even a weaker stance on the legitimate extent of state intervention may be troubling.

The historic British approach to looking after the welfare of children is to treat this as entirely the business of families, and for outside agencies to become involved only when problems show up that are so serious they require outside intervention. This approach is highly expensive, and does not work. By failing to identify potential problems before they happen, or immediately they begin to happen, situations of potential stress and maltreatment are allowed to grow and develop until they become very serious. (Loughton 2015, 17)

Here, although there is no suggestion that certain people should not procreate, there is advocacy for the intervention of the state into the family in cases where the former deems parenting inadequate. While the importance of protecting child welfare and ensuring equitable life chances cannot be disputed, the position advanced is problematic if state responsibility is not constrained by other ethically important factors.

## DISCUSSION

The preceding analysis explored some of the key theoretical and practical ethical implications of contemporary EI policy guidance in the UK. We applied a critical philosophical lens to show where the policies are unclear or overly idealistic; in doing so, we have demonstrated several problems with the arguments underlying policy proposals. However, although the guidance is deficient in certain respects, this does not mean that the proposals are useless or to be disregarded. Rather, we suggest that our analysis underwrites the importance of philosophical and bioethical scrutiny of public policy, as this can help to show where it is in need of modification before being implemented. Unless we closely interrogate the underlying values and assumptions in ostensibly “obvious” policy initiatives, we run the danger of subverting the plurality of values and political outlooks held by the society in which policies are to be implemented.

We should not expect academic philosophical rigor from policy guidance. Though policymakers and bioethicists often share the goal of creating a better society, their roles, methods, and skills are different. Put simply, while the value of a bioethicist’s work is measured at least in part in terms of how well she negotiates conflicting theories to form a logically consistent argument, policy is not evaluated in the same way; arguably, the final policy recommendation is more important than the process of arriving at this conclusion. As Chan notes:

In the policy context, it is sometimes less important to be absolutely precise or ‘correct’ … in our arguments than to be convincing in order to achieve the desired outcome. If what we care about are consequences, then, in some cases, the means of less than perfect moral reasoning may be justified by the ends of a functional policy that achieves the best outcome. (Chan 2015, 9)

Policies must function ethically in the real world, and since policymaking is a political process it necessarily involves compromise, attempting to provide solutions that are maximally but imperfectly pleasing to as many stakeholders as possible. It is therefore perhaps unsurprising that policymakers adopt a “no blame” narrative of parenting, even if this has problematic ethical implications. The government seeks to represent and serve the interests of all citizens, and blaming or stigmatizing a certain group may be counterproductive in this respect.

With this in mind, it is unfair and unhelpful for philosophers or bioethicists to work merely as “technicians,” providing a “logic monitoring” service for policymakers (Kymlicka 1993, 9). Rather, good ethical analysis should not simply point out logical flaws but explore what the practical implications of inconsistencies in reasoning might be. One reason for exposing weaknesses in policy proposals is because it is important to distinguish between policies that are genuinely well designed and—all things considered—applicable to the conditions of society, and specious policies that have the ring of truth, perhaps because of their appeal to an especially emotive issue such as child welfare, but that have ethically problematic implications in practice.

Bioethicists are well placed to provide a critical eye that is sensitive to such concerns. Though not all bioethicists are philosophers, the ethical analysis undertaken here is philosophical, drawing as it does from philosophical literatures and focusing on the quality of argument found within the policy corpus. It is ironic that a criticism frequently leveled at this kind of philosophical analysis is that it is idealistic and lacks practical application; indeed, as Hook (1980, 470) notes, given the nature of their work, philosophers in particular “are likely to be more aware than others of the disparities between the ideal and the actual.” While bioethicists can use the idea of a green or white paper to defend themselves against the charge of overly abstracted concerns (Oswald 2013), policymakers do not have the same luxury; creating implementable real-world solutions is their purpose. Therefore, though it is unfair to require philosophical rigor of policymakers, it is reasonable to insist that their policies are sufficiently coherent as to be implementable, and it is in assessing this that philosophical scrutiny can add value. This is one reason why we have focused upon responsibility throughout our analysis, by virtue of its central relevance to the particular social challenges to be met in the context of EI. We argue that a reasonable level of clarity is required in this regard, since if policy is to successfully create change that improves outcomes for children, then relevant stakeholders must understand who is to be held responsible for what. Otherwise, individuals and groups may always assume that someone else is responsible, and so in practice, no action is taken since no one can be held to account.

Further, policy aims to represent and serve the best interests of society at large. In this respect we suggest that the documents reveal another weakness: namely, they fail to adequately address the interests of all key stakeholders, and in particular, often neglect the interests of parents. Kymlicka’s analysis of the role that moral philosophy might play in policy development is illuminating here. He argues that while policymakers should not be expected to adhere to specific moral theories, nevertheless it is imperative that they “look at things morally.” He defines this as a form of respect for persons, whereby policymakers should consider interests as widely as possible:

[Policymakers should] take people’s lives and interests seriously [and] consider people’s interests with empathy. [Policymakers] can consider the fate of the weak and marginalized, as well as the legitimate interests of the more vocal and powerful. A responsible commission will do what it can to put itself in the shoes of all those who are affected … to take those impacts into account in its recommendations, and seek creative policies that accommodate them wherever possible. (Kymlicka 1993, 15)

In our context we revealed that, for example, by exploiting a seemingly liberal and compassionate “no blame” narrative that sees parents as the victims of circumstance, the policy documents are able to advance proposals that, under examination, actually pay scant regard to the justified interests some parents have in autonomy and freedom from interference from the state. Consequently, we argue that EI policymakers should ensure that they are even-handed in their approach, guided by the principle that social policies are ones in which everyone, and not only the disadvantaged, has a stake.

## CONCLUSION

Our aim was to conduct an ethical analysis of EI policy documents, focusing on the concept of responsibility. We have argued that while we cannot expect fully realized and watertight theoretical accounts of justice from policymakers, we can at the least demand a reasonably clear account of responsibility in practice: one that is sufficiently coherent as to be implementable, given the conditions of society.

Having said this, we acknowledge that creating a coherent practical account of responsibility is challenging. Given that philosophers cannot agree upon an account of moral responsibility in abstraction, it is perhaps to be expected that policymakers have difficulty advancing a fully coherent account of responsibility that is workable in the real world. However, we should not give up. Philosophical scrutiny of the kind conducted here can help policymakers to improve their policies by bringing to light the potential implications of holding certain views, highlighting philosophical literatures that might be helpful, revealing where policy is overly rhetorical and lacks clarity, and ultimately aiming for a policy that has traction and can be implemented. As such, our analysis should not be seen as a criticism of policymakers, but rather as an offer of assistance in what is a difficult task. Philosophers and bioethicists have skills that are valuable to policymakers, and equally there is much that our discipline can learn from those involved in political decision making in the “real world.” We therefore hope to have shown that the kind of philosophical analysis conducted here is useful to policymakers seeking to make society fairer through EI, and may lead to productive collaborations in future.

## Footnotes

1. For a list of all EI programs that have been evaluated by the Early Intervention Foundation, see The EIF Guidebook, available online at http://guidebook.eif.org.uk

2. It should be noted that other EI programs are not necessarily state funded, and many are run by charities or not-for-profit organisations. However, we focus predominantly upon government-funded EI programs here, because of the central goal of this article, that is, to provide an ethical analysis of UK EI policy guidance, which necessarily involves the relationship of the state’s responsibility to intervene in the lives of parents and children.

3. Michael Marmot is a relative outlier in this respect, since his research focuses on the social determinants of health. Marmot’s research apparently shows that it is one’s relative position in society, rather than one’s absolute income or other social factors that is causally responsible for poor health in many cases

4. There is a very large philosophical literature on causal and moral responsibility, which we do not have space to discuss here. For a helpful summary, see Eshleman (2016).

5. It is important to note that lack of knowledge is not obviously enough to discharge moral responsibility in all cases; this is made clear in both the philosophical and legal literature. It usually matters how one came to be in a position of insufficient knowledge, and what actions one can reasonably take to gain the required knowledge (Zimmerman 1997). We do not have the space to explore parental culpable ignorance here, except to point out that such lack of knowledge typically results in what is termed neglect rather than abuse, since in the former case “the carer is without motive and unaware of the damage being caused” (Golden et al. 2003).

6. Here we say “made probable” and “more likely” because the scientific literature suggests that upbringing in the early years is just one factor that influences or shapes an individual’s character and outcomes in late life. However, EI policy often makes such claims using stronger language, suggesting that early experiences determine a child’s later life outcomes. The debate about “parental determinism” has been discussed elsewhere: see, for example, Lowe and colleagues (Lowe et al. 2015).

7. An exploration of the large literature on various theories of distributive justice is beyond the scope of this article. See Lamont and Cavor (2017).

8. In fact, Cassidy takes seriously the position she espouses in the paper, and it appears stronger than just a thought experiment. Nevertheless, it is not policy guidance, and in the contemporary UK context at least it is hard to imagine that any policy guidance that was to advance this view adducing her argument in support of it could be implemented.
